# Type 2 diabetes is an independent predictor of lowered peak aerobic capacity in heart failure patients with non-reduced or reduced left ventricular ejection fraction

**DOI:** 10.1186/s12933-020-01114-4

**Published:** 2020-09-19

**Authors:** Takahiro Abe, Takashi Yokota, Arata Fukushima, Naoya Kakutani, Takashi Katayama, Ryosuke Shirakawa, Satoshi Maekawa, Hideo Nambu, Yoshikuni Obata, Katsuma Yamanashi, Ippei Nakano, Shingo Takada, Isao Yokota, Koichi Okita, Shintaro Kinugawa, Toshihisa Anzai

**Affiliations:** 1 Department of Cardiovascular Medicine, Faculty of Medicine and Graduate School of Medicine, Hokkaido University, Kita-15 Nishi-7, Kita-Ku, Sapporo, 060-8638 Japan; 2 Department of Biostatistics, Faculty of Medicine and Graduate School of Medicine, Hokkaido University, Sapporo, Japan; 3 Graduate School of Lifelong Sport, Hokusho University, Ebetsu, Japan

**Keywords:** Exercise, Heart failure, Oxygen uptake, Type 2 diabetes

## Abstract

**Background:**

Although type 2 diabetes mellitus (T2DM) is one of the most frequent comorbidities in patients with chronic heart failure (CHF), the effects of T2DM on the exercise capacity of CHF patients are fully unknown. Here, we tested the hypothesis that the coexistence of T2DM lowers CHF patients’ peak aerobic capacity.

**Methods:**

We retrospectively analyzed the cases of 275 Japanese CHF patients with non-reduced ejection fraction (left ventricular ejection fraction [LVEF] ≥ 40%) or reduced EF (LVEF < 40%) who underwent cardiopulmonary exercise testing. We divided them into diabetic and nondiabetic groups in each CHF cohort.

**Results:**

The mean peak oxygen uptake (VO_2_) value was 16.87 mL/kg/min in the non-reduced LVEF cohort and 15.52 mL/kg/min in the reduced LVEF cohort. The peak VO_2_ was lower in the diabetics versus the nondiabetics in the non-reduced LVEF cohort with the mean difference (95% confidence interval [95% CI]) of − 0.93 (− 1.82 to − 0.04) mL/kg/min and in the reduced LVEF cohort with the mean difference of − 1.05 (− 1.96 to − 0.15) mL/kg/min, after adjustment for age-squared, gender, anemia, renal function, LVEF, and log B-type natriuretic peptide (BNP). The adjusted VO_2_ at anaerobic threshold (AT), a submaximal aerobic capacity, was also decreased in the diabetic patients with both non-reduced and reduced LVEFs. Intriguingly, the diabetic patients had a lower adjusted peak O_2_ pulse than the nondiabetic patients in the reduced LVEF cohort, but not in the non-reduced LVEF cohort. A multivariate analysis showed that the presence of T2DM was an independent predictor of lowered peak VO_2_ in CHF patients with non-reduced LVEF and those with reduced LVEF.

**Conclusions:**

T2DM was associated with lowered peak VO_2_ in CHF patients with non-reduced or reduced LVEF. The presence of T2DM has a negative impact on CHF patients’ exercise capacity, and the degree of impact is partly dependent on their LV systolic function.

## Background

Exercise intolerance is one of the cardinal manifestations of chronic heart failure (CHF), and it influences the disease severity and prognosis of individuals with CHF. In particular, a lowered peak oxygen uptake (VO_2_) is known as a strong predictor of all-cause mortality in CHF patients [1]. We and others have demonstrated that skeletal muscle dysfunction plays a crucial role in exercise intolerance in CHF patients [2, 3]. Among the established risk factors for CHF, type 2 diabetes mellitus (T2DM) is one of the most frequently observed comorbidities of CHF. The survival rate of CHF patients with T2DM is markedly reduced compared to those without type 2 diabetes [4, 5]. Intriguingly, a common phenotype of patients with T2DM is the presence of one or more skeletal muscle alterations such as impaired energy metabolism and muscle fiber-type switch [6, 7], and this phenotype is similar to that of patients with CHF. In addition, lowered aerobic capacity independently predicts the all-cause mortality of diabetic patients [8]. Accordingly, T2DM may further reduce the aerobic capacity (including the peak VO_2_) of CHF patients.

Several research groups have shown that T2DM has negative effects on the exercise capacity of CHF patients who have a reduced left ventricular ejection fraction (LVEF < 40%) [9, 10] or a preserved LVEF (≥ 50%) [11]. However, there was no study that comprehensively investigated effects of T2DM on the exercise capacity in CHF patients with non-reduced LVEF (≥ 40%) and those with reduced LVEF (< 40%). Herein, we examined whether T2DM is an independent predictor of lowered peak VO_2_ in CHF patients with either non-reduced or reduced LVEF, and whether the presence of T2DM has a differential impact on exercise capacity between CHF patients with non-reduced LVEF and those with reduced LVEF.

## Methods

### Patients

We retrospectively analyzed the cases of a total of 275 stable Japanese patients with CHF who underwent cardiopulmonary exercise testing (CPET) between January 2009 and March 2016 at Hokkaido University Hospital. Before the CPET, all of the patients had a history of one or more hospital admissions due to worsening HF diagnosed on the basis of the American College of Cardiology Foundation (ACCF)/American Heart Association (AHA) Task Force on Practice guidelines [12]. In particular, patients who had a normal LVEF at the time of hospitalization was diagnosed as HF when they met the following criteria; (1) clinical signs or symptoms of HF; (2) evidence of abnormal LV diastolic function determined by echocardiography or cardiac catheterization; (3) exclusion of other potential noncardiac causes. All the patients were at stage C or stage D HF in the ACC/AHA guidelines. T2DM was defined as a fasting blood glucose level ≥ 126 mg/dL, a glycohemoglobin A1c (HbA1c) level ≥ 6.5%, and/or the need for an oral hypoglycemic agent or insulin. Patients who were unable to perform maximal exercise due to pulmonary disease, peripheral artery disease, stroke, or orthopedic disease were excluded. We also excluded patients in whom the peak respiratory exchange ratio (RER) did not reach 1.05 in the CPET, as they might not be able to perform maximal exercise. The present investigation is a part of a large cohort study that used the database of the Exercise Testing Laboratory at Hokkaido University Hospital, and thus, some of the data are from the same patients whose data have been published in a different context [13].

### Cardiopulmonary exercise testing

Each of the patients exercised on an upright cycle ergometer (Aerobike 75XLII, Combi Wellness, Tokyo, Japan) using a ramp protocol (5–25 watts/min). A respiratory gas analysis was simultaneously performed with a breath-by-breath apparatus (Aeromonitor AE300S, Minato Medical Science, Osaka, Japan) as described [14]. Peak VO_2_ was defined as the maximal VO_2_ attained during the symptom-limited incremental exercise, and the anaerobic threshold (AT) was determined by the V-slope method [15]. The RER was calculated as the ratio of carbon dioxide production (VCO_2_) to VO_2_. We calculated the ΔVO_2_/Δworkload as the ratio of the peak VO_2_ to the peak workload. The peak O_2_ pulse was calculated as the ratio of the peak VO_2_ to the peak heart rate (HR). We defined HR reserve as: HR reserve = peak HR − resting HR. The chronotropic index (CI) was defined as: CI = (peak HR − resting HR) / (predicted maximal HR − resting HR), where the predicted maximal HR was calculated as: the predicted maximal HR = 220 − age. The lowest minute ventilation (VE)/VCO_2_ during exercise was evaluated to assess patients’ ventilatory efficiency.

### Other clinical variables and outcomes

We reviewed the patients’ medical records to collect their demographic and clinical data including age, gender, body mass index (BMI), New York Heart Association (NYHA) functional class, primary cause of HF, cardiovascular risk factor(s), and medication(s). Each patient’s echocardiographic parameters and laboratory data were acquired within 30 days before or after he or she underwent the CPET. The left ventricular end-diastolic diameter (LVEDD) of each patient was evaluated from the parasternal long-axis view, and the LVEF was measured by the biplane method of disks using echocardiography. To assess LV diastolic function, we measured the peak early-diastolic and atrial systolic transmitral flow velocities (E and A, respectively) and the deceleration time of the E wave using pulsed-Doppler echocardiography, and the peak early-diastolic mitral annular velocity (e′) at the septal and lateral sides of the annulus with tissue-Doppler imaging. The e′ was averaged, and then, E/e′ was calculated. Hemoglobin, plasma B-type natriuretic peptide (BNP), and the estimated glomerular filtration rate (eGFR) were determined by routine in-house analyses. The eGFR was calculated from the serum creatinine values and age using the Japanese equation [16]: eGFR = 194 × (serum creatinine, mg/dL)^−1.094^ × (age, years)^−0.287^ × (0.739 if female).

### Statistical analyses

Data are presented as the mean ± standard deviation (SD) for continuous variables and as numbers (percentages) for categorical variables. We divided the CHF patients into diabetic and nondiabetic groups based on the presence/absence of T2DM. We also conducted a subgroup analysis defined by the patients’ non-reduced LVEF (LVEF ≥ 40%) or reduced LVEF (LVEF < 40%). We compared the CPET parameters between the diabetic and nondiabetic groups in each subgroup after adjustment for age-squared, gender, hemoglobin, eGFR, LVEF, and log BNP, all of which are considered confounders. We performed a multivariate analysis to determine the independent variables of peak VO_2_ in the CHF patients, including T2DM (treated as the presence of T2DM = 1; the absence of T2DM = 0), LVEF, log BNP, age-squared, gender, hemoglobin, and eGFR. All analyses were performed using the JMP 12.2.0 program (SAS Institute, Cary, NC). A confidence level was set at 95%. Probability (*P*)-values < 0.05 were considered significant.

## Results

### The baseline characteristics of the total CHF population

The baseline data of the total population of CHF are summarized in Table 1. Of the 275 patients with CHF, 78 patients (28%) had T2DM. The mean age of the total CHF population was 56 years (61 years in the diabetic group and 54 years in the nondiabetic group). The primary cause of HF was ischemic heart disease (21% of the total population; 28% of the diabetics and 19% of the nondiabetics), dilated cardiomyopathy (26% of the total population; 27% of the diabetics and 26% of the nondiabetics), or other causes including hypertrophic cardiomyopathy, hypertensive heart disease, and valvular heart disease. In addition, 48% of the total CHF population had a non-reduced LVEF.

**Table 1:** Baseline characteristics of the total CHF population

	All (n = 275)	Diabetic (n = 78)	Nondiabetic (n = 197)	*P*-value
Demographic findings				
Age, years	56 ± 16	61 ± 12	54 ± 17	< 0.01
Male	212 (77%)	63 (81%)	149 (76%)	0.36
BMI, kg/m^2^	23.4 ± 4.5	24.9 ± 5.9	22.8 ± 3.7	0.01
NYHA functional class				0.27
I	44 (16%)	9 (12%)	35 (18%)	
II	176 (64%)	50 (64%)	126 (64%)	
III	54 (20%)	19 (24%)	35 (18%)	
Primary cause of HF				
Ischemic cause	59 (21%)	22 (28%)	37 (19%)	0.09
Dilated cardiomyopathy	72 (26%)	21 (27%)	51 (26%)	0.86
Others	144 (52%)	35 (45%)	109 (55%)	0.12
Hypertension	103 (37%)	40 (51%)	63 (32%)	< 0.01
Dyslipidemia	103 (37%)	45 (58%)	58 (29%)	< 0.01
Atrial fibrillation	65 (24%)	27 (35%)	38 (19%)	< 0.01
Echocardiographic findings				
LVEDD, mm	60.0 ± 10.8	60.3 ± 9.0	59.9 ± 11.4	0.66
LVEF, %	40.6 ± 14.5	39.3 ± 11.5	41.1 ± 15.5	0.69
E/A ratio^a^	1.23 ± 0.88	1.33 ± 1.04	1.20 ± 0.83	0.84
Deceleration time^b^, ms	208.5 ± 64.0	202.4 ± 57.8	210.8 ± 66.3	0.35
E/e′^c^	10.9 ± 4.4	12.2 ± 4.8	10.5 ± 4.1	< 0.01
Laboratory measurements				
Hemoglobin, g/dL	13.3 ± 1.7	13.2 ± 1.9	13.3 ± 1.7	0.65
Serum creatinine, mg/dL	1.04 ± 0.42	1.20 ± 0.54	0.98 ± 0.34	< 0.01
eGFR, mL/min/1.73 m^2^	63.4 ± 22.5	54.4 ± 18.8	67.0 ± 22.9	< 0.01
HbA1c, %	5.9 ± 0.8	6.7 ± 0.9	5.6 ± 0.4	< 0.01
BNP, pg/mL	291.6 ± 405.8	321.0 ± 421.8	280.5 ± 400.3	0.07
Medications:				
ACE inhibitors or ARBs	229 (83)	73 (94)	156 (79)	< 0.01
β blockers	220 (80)	70 (91)	150 (76)	< 0.01
MRAs	136 (49)	40 (52)	95 (48)	0.58
Statins	104 (38)	39 (51)	65 (33)	< 0.01
Insulin	7 (3)	7 (9)	–	NA
Metformin	11 (4)	11 (14)	–	NA
DPP4 inhibitors	14 (5)	14 (18)	–	NA
Sulfonylureas	11 (4)	11 (14)	–	NA

Data are mean ± SD or n (%). ‘Ischemic cause’ indicates coronary artery disease or myocardial infarction

A, peak velocity of mitral inflow during atrial systole; ACE, angiotensin converting enzyme; ARB, angiotensin II receptor blocker; BMI, body mass index; BNP, B–type natriuretic peptide; DPP4, dipeptidyl peptidase 4; E, peak velocity of mitral inflow during early diastole; e’, average of septal and lateral mitral annular early diastolic peak velocities; eGFR, estimated glomerular filtration rate; HbA1c, glycohemoglobin A1c; NA, not applicable; LVEDD, left ventricular end–diastolic diameter; LVEF, left ventricular ejection fraction; MRAs, mineralocorticoid receptor antagonists; NYHA, New York Heart Association

^a^ n = 169, 38, 131 (all, diabetic, nondiabetic)

^b^ n = 190, 55, 145 (all, diabetic, nondiabetic)

^c^ n = 189, 54, 135 (all, diabetic, nondiabetic)

As expected, the HbA1c level was higher in the diabetic patients than in the nondiabetic patients. Renal function was impaired in the diabetic patients with higher serum creatine and lower eGFR compared to the nondiabetic patients. The mean level of plasma BNP, a marker of the severity of HF, was 291.6 pg/dL in the total CHF population, 321.0 pg/dL in the diabetic patients with CHF, and 280.5 pg/dL in the nondiabetic patients with CHF. Regarding medications, 83% of the total CHF population were being treated with an angiotensin converting enzyme (ACE) inhibitor or angiotensin receptor blocker (ARB), and 80% received β-blockers; 9% of the diabetic patients with CHF were treated with insulin.

### The baseline characteristics of the CHF patients with non-reduced or reduced LVEF

We divided the total CHF population into two subgroups: the patients with non-reduced LVEF (≥ 40%; n = 131) and the patients with reduced LVEF (< 40%; n = 144). The baseline data of each subgroup are summarized in Table 2. The mean ages of the CHF patients with LVEF ≥ 40% and those with LVEF < 40% were 58 years and 54 years, and 67% and 86% of these subgroups were male, respectively. The ratio of New York Heart Association (NYHA) class I (asymptomatic)/class II (mild CHF)/class III (moderate-to-severe CHF) was 22%/68%/10% in the LVEF ≥ 40% group and 10%/61%/28% in the LVEF < 40% group. The E/e’ was higher in the diabetics compared to the nondiabetics in the non-reduced LVEF cohort, indicating impaired LV diastolic function in the diabetic patients with non-reduced LVEF. The mean level of plasma BNP was 180.0 pg/dL in the LVEF ≥ 40% and higher at 383.7 pg/dL in the LVEF < 40% group. Diabetic patients with non-reduced LVEF were more often taking ACE inhibitors or ARBs and β-blockers, while those with reduced LVEF were more often taking statins.

**Table 2:** Baseline characteristics of the CHF patients with non-reduced LVEF (≥ 40%) or reduced LVEF (< 40%)

	LVEF ≥ 40%				LVEF < 40%			
	All (n = 131)	Diabetic (n = 34)	Nondiabetic (n = 97)	*P*-value	All (n = 144)	Diabetic (n = 44)	Nondiabetic (n = 100)	*P*-value
Demographic finding								
Age, years	58 ± 16	63 ± 11	57 ± 17	0.05	54 ± 16	60 ± 13	52 ± 17	0.01
Male	88 (67%)	25 (74%)	63 (65%)	0.36	124 (86%)	38 (86%)	86 (86%)	0.95
BMI, kg/m^2^	23.6 ± 4.0	25.2 ± 5.3	23.1 ± 3.3	< 0.01	23.2 ± 4.9	24.8 ± 6.4	22.6 ± 3.9	0.22
NYHA functional class				0.69				0.31
I	29 (22%)	6 (18%)	23 (24%)		15 (10%)	3 (7%)	12 (12%)	
II	89 (68%)	25 (73%)	64 (66%)		88 (61%)	25 (57%)	63 (63%)	
III	13 (10%)	3 (9%)	10 (10%)		41 (28%)	16 (36%)	25 (25%)	
Primary cause of HF								
Ischemic cause	25 (19%)	8 (24%)	17 (18%)	0.44	34 (24%)	14 (32%)	20 (20%)	0.12
Dilated cardiomyopathy	17 (13%)	4 (12%)	13 (13%)	0.81	55 (38%)	17 (39%)	38 (38%)	0.94
Others	90 (69%)	23 (68%)	67 (69%)	0.88	54 (38%)	12 (28%)	42 (42%)	0.09
Hypertension	60 (46%)	24 (71%)	36 (37%)	< 0.01	43 (30%)	16 (36%)	27 (27%)	0.26
Dyslipidemia	48 (37%)	20 (59%)	28 (29%)	< 0.01	55 (38%)	25 (57%)	30 (30%)	< 0.01
Atrial fibrillation	33 (25%)	14 (41%)	19 (20%)	0.01	32 (22%)	13 (30%)	19 (19%)	0.16
Echocardiographic findings								
LVEDD, mm	53.5 ± 9.1	56.0 ± 7.7	52.6 ± 9.4	0.04	64.8 ± 9.3	63.1 ± 8.8	65.5 ± 9.5	0.47
LVEF, %	53.1 ± 10.0	49.9 ± 7.7	54.2 ± 10.5	0.06	29.2 ± 6.1	31.1 ± 5.7	28.4 ± 6.2	0.02
E/A ratio^a^	1.07 ± 0.54	1.24 ± 0.98	1.03 ± 0.4	0.89	1.33 ± 1.03	1.37 ± 1.09	1.32 ± 1.02	0.85
Deceleration time^b^, msec	235.3 ± 71.1	221.6 ± 72.0	239.8 ± 70.9	0.26	190.3 ± 51.5	191.5 ± 45.4	189.8 ± 54.1	0.73
E/e′^c^	10.2 ± 3.5	11.7 ± 3.8	9.6 ± 3.2	0.03	11.5 ± 4.8	12.5 ± 5.4	11.0 ± 4.5	0.11
Laboratory measurements								
Hemoglobin, g/dL	13.3 ± 1.6	13.3 ± 1.8	13.3 ± 1.6	0.87	13.3 ± 1.8	13.2 ± 2.0	13.4 ± 1.7	0.48
Serum creatinine, mg/dL	1.0 ± 0.3	1.1 ± 0.4	0.9 ± 0.3	0.03	1.1 ± 0.5	1.3 ± 0.6	1.0 ± 0.4	< 0.01
eGFR, mL/min/1.73 m^2^	64.7 ± 22.2	57.0 ± 20.0	67.4 ± 22.4	0.02	62.2 ± 22.8	52.4 ± 17.9	66.6 ± 23.4	< 0.01
HbA1c, %	5.9 ± 0.8	6.8 ± 0.9	5.5 ± 0.4	< 0.01	5.9 ± 0.7	6.6 ± 0.9	5.6 ± 0.4	< 0.01
BNP, pg/mL	180.0 ± 299.1	192.0 ± 174.5	176.1 ± 330.5	0.03	383.7 ± 457.2	409.1 ± 512.2	373.0 ± 434.3	0.66
Medications								
ACE inhibitors or ARBs	94 (72)	30 (88)	64 (66)	0.01	135 (94)	43 (98)	92 (92)	0.19
β blockers	84 (65)	27 (82)	57 (59)	0.02	136 (94)	43 (98)	93 (93)	0.25
MRAs	41 (32)	13 (39)	28 (29)	0.26	94 (65)	27 (61)	67 (67)	0.51
Statins	48 (37)	15 (46)	33 (34)	0.24	56 (39)	24 (55)	32 (32)	0.01
Insulin	2 (2)	2 (6)	–	NA	5 (3)	5 (12)	–	NA
Metformin	4 (3)	4 (12)	–	NA	7 (5)	7 (16)	–	NA
DPP4 inhibitors	7 (5)	7 (21)	–	NA	7 (5)	7 (16)	–	NA
Sulfonylureas	7 (5)	7 (22)	–	NA	4 (3)	4 (9)	–	NA

Data are mean ± SD or n (%). Abbreviations are as defined in Table 1

^a^n = 66, 12, 54 (all, diabetic, nondiabetic in LVEF ≥ 40%) and n = 103, 26, 77 (all, diabetic, nondiabetic in LVEF < 40%)

^b^n = 81, 20, 61 (all, diabetic, nondiabetic in LVEF ≥ 40%) and n = 109, 35, 84 (all, diabetic, nondiabetic in LVEF < 40%)

^c^n = 75, 20, 55 (all, diabetic, nondiabetic in LVEF ≥ 40%) and n = 114, 34, 80 (all, diabetic, nondiabetic in LVEF < 40%)

We further divided the non-reduced LVEF cohort into the preserved LVEF (≥ 50%; n = 73) and the mid-range LVEF cohorts (40–49%; n = 58), which is summarized in Additional file 1: Table S1. Diabetic patients with LVEF ≥ 50% had more often hypertension and atrial fibrillation, while those with LVEF 40–49% had more often hypertension and dyslipidemia. The E/e′ was higher in the diabetics in the preserved LVEF cohort.

### The exercise capacity of the CHF patients with non-reduced or reduced LVEF

The CPET data of the CHF patients with non-reduced or reduced LVEF are summarized in Table 3. The mean peak VO_2_ value was 16.87 mL/kg/min in the LVEF ≥ 40% group and 15.52 mL/kg/min in the LVEF < 40% group. After the adjustment for age-squared, gender, anemia, renal function, LVEF, and log BNP, the peak VO_2_ was lower in the diabetics compared to the nondiabetics among the patients with LVEF ≥ 40%, with the mean difference (95% CI) of − 0.93 (− 1.82 to − 0.04) mL/kg/min; the peak VO_2_ was also lower in the diabetics compared to the nondiabetics among the patients with LVEF < 40%, with the mean difference of − 1.05 (− 1.96 to − 0.15) mL/kg/min. The O_2_ pulse (i.e., the O_2_ consumed per beat) at peak exercise was decreased in the diabetics compared to the nondiabetics only in the LVEF < 40% group. The AT VO_2_, a marker of the body’s submaximal aerobic capacity, was also lower in the diabetics than in the nondiabetics in the LVEF ≥ 40% group, with the adjusted mean difference of − 0.65 (− 1.18 to − 0.12) mL/kg/min, and in the LVEF < 40% group with the adjusted mean difference of − 0.66 (− 1.11 to − 0.21) mL/kg/min. In contrast, there was no difference in the lowest VE/VCO_2_ between the diabetics and nondiabetics in either LVEF group. When we further divided the non-reduced LVEF cohort into the preserved LVEF and the mid-range LVEF cohorts, the peak VO_2_ and the AT VO_2_ were lowered in the diabetics in the LVEF 40-49% group, but not in the LVEF ≥ 50% group, after adjustment for age-squared, gender, anemia, renal function, LVEF, and log BNP (Additional file 1: Table S2).

**Table 3:** Cardiopulmonary exercise testing parameters of the CHF patients with non-reduced LVEF (≥ 40%) or reduced LVEF (< 40%)

	LVEF ≥ 40%					LVEF < 40%				
	All (n = 131)	Diabetic (n = 34)	Nondiabetic (n = 97)	Unadjusted mean difference (95% CI)	Adjusted mean difference (95% CI)	All (n = 144)	Diabetic (n = 44)	Nondiabetic (n = 100)	Unadjusted mean difference (95% CI)	Adjusted mean difference (95% CI)
Peak VO_2_, mL/kg/min	16.87 ± 4.98	15.16 ± 3.82	17.46 ± 5.22	− 1.15* (− 2.12 to − 0.18)	− 0.93* (− 1.82 to − 0.04)	15.52 ± 5.17	13.46 ± 3.94	16.43 ± 5.41	− 1.48* (− 2.38 to − 0.59)	− 1.05* (− 1.96 to − 0.15)
Peak workload, watts	91.0 ± 40.2	90.1 ± 43.2	91.4 ± 39.3	− 0.6 (− 8.6 to 7.4)	− 0.2 (− 6.6 to 6.3)	84.2 ± 34.1	76.9 ± 27.8	87.3 ± 36.1	− 5.2 (− 11.3 to 0.9)	− 2.5 (− 8.0 to 3.1)
Peak HR, beats/min	123.8 ± 30.7	116.5 ± 29.8	126.4 ± 30.7	− 5.0 (− 11.0 to 1.1)	− 2.4 (− 8.7 to 4.0)	120.7 ± 28.8	116.0 ± 24.5	122.8 ± 30.4	− 3.4 (− 8.5 to 1.7)	3.5 (− 2.0 to 9.0)
Peak RER	1.24 ± 0.13	1.19 ± 0.12	1.26 ± 0.13	− 0.04* (− 0.06 to − 0.01)	− 0.04* (− 0.06 to − 0.01)	1.26 ± 0.12	1.28 ± 0.13	1.25 ± 0.11	0.01 (− 0.01 to 0.04)	0.02 (− 0.01 to 0.04)
ΔVO_2_/Δworkload	7.21 ± 2.09	6.79 ± 1.99	7.35 ± 2.11	− 0.28 (− 0.69 to 0.13)	− 0.13 (− 0.54 to 0.27)	7.04 ± 1.90	6.65 ± 1.78	7.21 ± 1.94	− 0.28 (− 0.62 to 0.07)	− 0.29 (− 0.65 to 0.07)
Peak O_2_ pulse, mL/beats	8.77 ± 3.02	9.28 ± 3.28	8.59 ± 2.92	0.34 (− 0.25 to 0.94)	0.21 (− 0.23 to 0.66)	8.52 ± 2.90	7.98 ± 2.88	8.75 ± 2.89	− 0.38 (− 0.90 to 0.13)	− 0.75* (− 1.24 to − 0.25)
HR reserve, beats/min	55.8 ± 26.2	49.1 ± 25.3	58.2 ± 26.3	− 4.6 (− 9.7 to 0.6)	− 3.2 (− 8.8 to 2.3)	51.5 ± 25.3	47.9 ± 22.3	53.2 ± 2 6.4	− 2.7 (− 7.2 to 1.9)	1.7 (− 3.4 to 6.9)
Chronotropic index	0.60 ± 0.29	0.57 ± 0.33	0.62 ± 0.28	− 0.02 (− 0.08 to 0.04)	− 0.02 (− 0.08 to 0.05)	0.54 ± 0.27	0.52 ± 0.24	0.55 ± 0.28	− 0.01 (− 0.06 to 0.03)	0.03 (− 0.03 to 0.08)
AT VO_2_, mL/kg/min	10.75 ± 2.74	9.79 ± 1.59	11.09 ± 2.97	− 0.65* (− 1.18 to − 0.12)	− 0.65* (− 1.21 to − 0.08)	9.76 ± 2.62	8.78 ± 2.15	10.19 ± 2.70	− 0.71* (− 1.16 to − 0.25)	− 0.66* (− 1.11 to − 0.21)
Lowest VE/VCO_2_	33.6 ± 6.1	34.0 ± 4.4	33.4 ± 6.6	0.3 (− 0.9 to 1.5)	0.2 (− 0.9 to 1.4)	36.3 ± 8.2	37.9 ± 8.9	35.6 ± 7.8	1.1 (− 0.3 to 2.6)	0.6 (− 1.0 to 2.1)

Data are mean ± SD. Mean difference between the diabetic and the nondiabetic in each CHF cohort is adjusted by age-squared, gender, hemoglobin, eGFR, LVEF, and log BNP

AT, anaerobic threshold; HR, heart rate; RER, respiratory exchange ratio; VCO_2_, carbon dioxide production; VE, minute ventilation; VO_2_, oxygen uptake; 95% CI, 95% confidence interval

* *P *< 0.05 vs. nondiabetics

### Predictors of lowered peak aerobic capacity in CHF patients with non-reduced or reduced LVEF

The results of the multivariate analysis revealed that after the adjustment for age-squared, gender, hemoglobin, eGFR, LVEF, and log BNP, the presence of T2DM was an independent predictor of lowered peak VO_2_ in the CHF patients with LVEF ≥ 40% and in those with LVEF < 40% (Table 4). The LVEF, but not log BNP, was also an independent variable to predict the peak VO_2_ only in the CHF patients with LVEF < 40% (Table 4). In the subgroup analysis of the LVEF ≥ 50% and LVEF 40–49% cohorts, the presence of T2DM was an independent predictor of lowered peak VO_2_ only in the LVEF 40-49% cohort (Additional file 1: Table S3).

**Table 4:** Multivariable analysis for peak VO_2_ in the CHF patients with non-reduced LVEF (≥ 40%) or reduced LVEF (< 40%)

	LVEF ≥ 40%		LVEF < 40%	
	Adjusted mean difference (mL/kg/min) 95% CI	*P*-value	Adjusted mean difference (mL/kg/min) 95% CI	*P*-value
Type 2 diabetes	− 0.93 (− 1.82 to − 0.04)	0.04	− 1.05 (− 1.96 to − 0.15)	0.02
LVEF, %	− 0.01 (− 0.10 to 0.09)	0.85	0.27 (0.14 to 0.40)	< 0.01
Log BNP	− 0.27 (− 1.61 to 1.08)	0.69	0.33 (− 1.31 to 1.97)	0.69

In addition to the variables displayed, age-squared, gender, hemoglobin, and eGFR are included in the analysis

## Discussion

Our retrospective analyses of 275 patients with CHF revealed that peak VO_2_ and AT VO_2_ were lower in the presence of T2DM in the patients with non-reduced LVEF as well as in those with reduced LVEF. Notably, the multivariate analysis showed that T2DM was an independent predictor of the lowered peak VO_2_ in both the CHF patients with non-reduced LVEF and those with reduced LVEF after the adjustment for age-squared, gender, hemoglobin, eGFR, LVEF, and log BNP, all of which may influence exercise capacity.

Exercise intolerance is a cardinal symptom of HF regardless of the LVEF [17]. It is widely recognized that comorbidity of T2DM may induce reduced cardiorespiratory fitness as well as worse clinical outcomes in patients with CHF. It has been demonstrated that T2DM lowers the peak VO_2_ in the CHF patients who have a reduced LVEF (< 40%) [9, 10] or a preserved LVEF (≥ 50%) [11]. To the best of our knowledge, the present study is the first to thoroughly investigate the negative effects of the presence of T2DM on the exercise capacity in CHF patients with both non-reduced and reduced LVEFs.

In the present study, the adjusted mean difference of peak VO_2_ between the diabetics and the non-diabetics was 0.93 mL/kg/min (approx. 6% of mean value of peak VO_2_ in all CHF cohort) and 1.05 mL/kg/min (approx. 7% of mean value of peak VO_2_ in all CHF cohort) in the patients with non-reduce LVEF and in those with reduced LVEF, respectively, and the difference of absolute value of peak VO_2_ between the diabetics and the nondiabetics appears to be small. However, a large clinical trial has reported that every 6% increase of peak VO_2_ after exercise training results in a 5% lower risk of all-cause mortality or all-cause hospitalization and an 8% lower risk of cardiovascular mortality or HF-related hospitalization in CHF patients [18]. Accordingly, a modest but significant difference of peak VO_2_ is clinically relevant in the prevention of HF development.

The VO_2_ is defined by the Fick equation: VO_2_ = cardiac output × arteriovenous O_2_ difference, where arteriovenous O_2_ difference is determined by peripheral factors. Low aerobic capacity may be generally caused by cardiac, vascular, or skeletal muscle dysfunction, which results in reduced O_2_ delivery or O_2_ utilization. Recent studies have shown that predominantly peripheral (i.e., noncardiac) factors contribute to reduced peak VO_2_ in CHF patients [19, 20]. In particular, skeletal muscle relies largely on O_2_ utilization for energy production during exercise, and it has been demonstrated that skeletal muscle dysfunction characterized by impaired mitochondrial oxidative capacity and fiber-type switch (i.e., a reduced ratio of slow oxidative fibers to fast glycolytic fibers) is a common feature of CHF patients with both preserved and reduced LVEFs [3, 21, 22]. Interestingly, the arteriovenous O_2_ difference at peak exercise is reported to be reduced in association with lowered peak VO_2_ in patients with T2DM [23], and a similar functional impairment of skeletal muscle is observed in patients with insulin resistance or T2DM [6, 7, 24]. Taken together, the presence of T2DM may further decrease the peak VO_2_ via skeletal muscle dysfunction in CHF patients with non-reduced or reduced LVEF.

On the other hand, we observed that the peak O_2_ pulse (a parameter that relies on the stroke volume at peak exercise) was lower in the diabetics than in the nondiabetics in the group of CHF patients with a reduced LVEF, but not in those with a non-reduced LVEF. In addition, the multivariate analysis showed that the LVEF was an independent predictor of lowered peak VO_2_ in the CHF patients with reduced LVEF. Accordingly, in patients with reduced LVEF, a decrease in LV systolic function (i.e., cardiac factors) may also underlie the association between T2DM and lowered peak aerobic capacity.

Another possible explanation of the lowered peak aerobic capacity in diabetic CHF patients is reduced muscle blood flow, which results in low O_2_ supply to skeletal muscle. Insulin resistance is associated with decreased vasodilatation and capillary recruitment [25], and a recent study revealed that peak VO_2_ was correlated with the muscle blood flow reserve in patients with T2DM [26]. In addition, microvascular complications of diabetes such as retinopathy and microalbuminuria are associated with a lowered peak VO_2_ in these patients [27]. Taken together, these findings suggest that the comorbidity of T2DM in CHF patients may have an additive effect that worsens the patients’ aerobic capacity via an impairment of endothelium-dependent vasodilatation.

Anemia, a determinant of lowered peak aerobic capacity, is known to be common in diabetic patients, and prevalence of anemia in diabetic patients could be doubled when they have an impaired renal function [28]. In addition, it has been shown that iron deficiency is frequently observed in diabetic patients with chronic kidney disease [29]. Since iron plays a role in O_2_ delivery, O_2_ storage, and muscle oxidative metabolism, iron deficiency can be an independent predictor of reduced peak VO_2_ in patients with CHF irrespective of hemoglobin levels [30]. In the present study, hemoglobin levels were comparable between the groups, but the eGFR was lower in the diabetics than in the non-diabetics in the CHF patients with non-reduced and reduced LVEFs. Although we did not measure serum ferritin levels, iron deficiency might affect lowered peak aerobic capacity in the CHF patients with T2DM.

Guazzi et al. demonstrated that the coexistence of T2DM reduces the peak VO_2_ in CHF patients with a reduced LVEF via an impairment of the alveolar-capillary gas transfer, possibly due to microangiopathy, which may lead to decreased O_2_ uptake through the lungs during exercise [9, 31]. In the present study, the lowest VE/VCO_2_, a parameter of ventilatory efficiency during exercise, did not differ between the CHF patients with and without T2DM, which is inconsistent with the previous report [9]. However, we did not evaluate the patients’ pulmonary function, and we therefore could not exclude the possibility of a contribution of the reduced capacity of pulmonary O_2_ perfusion to the depressed peak VO_2_ in CHF patients with T2DM.

Our analyses demonstrated that T2DM is an independent predictor of lowered peak VO_2_ in CHF patients with non-reduced LVEF (≥ 40%). However, in the multivariate analysis to determine the independent variables of peak VO_2_ after division of the non-reduced LVEF cohort into the preserved LVEF (≥ 50%) and the mid-range LVEF (40–49%) cohorts, the presence of T2DM did not reach statistical significance in the CHF patients with preserved LVEF, which is inconsistent with a previous study [11]. There are some possible reasons for it. First, in the present study, the number of CHF patients with preserved LVEF was small, and therefore, it may be insufficient to conduct multivariate analysis. Second, CHF with preserved LVEF is a complex, heterogenous syndrome involving multiple comorbidities such as hypertension and atrial fibrillation, which may weaken the impact of T2DM on the CHF patients’ exercise capacity. Accordingly, CHF patients with preserved LVEF is likely to be less susceptible to T2DM with respect to exercise capacity than those with reduced LVEF.

It has been shown that sedentary behavior and physical inactivity may induce exercise intolerance and increase risk of cardiovascular events in individuals with pre-diabetes or T2DM [32, 33] and in patients with CHF [34]. Accordingly, promotion of increased physical activity and exercise training is essential for CHF patients with T2DM to improve cardiorespiratory fitness and reduce cardiovascular or all-cause mortality. Indeed, various types of exercise training including moderate-intensity continuous training and high-intensity interval training are reported to improve cardiac function, vascular function, lipid profiles, and low-grade inflammation as well as exercise capacity in obese subjects or patients with T2DM [35–38] and in patients with CHF [39]. A large clinical trial has shown that sustained improvement of exercise capacity by intensive lifestyle intervention aiming at increased physical activity and weight loss reduces risk of incident HF in obese subjects with T2DM [40]. Because CHF patients with T2DM tend to have multiple comorbidities and optimal exercise training may vary depending on individual’s target of exercise parameters, types of exercise should be personalized [41]. For evaluation of exercise capacity, a 6-min walk test can be used as an alternative and cost-effective tool instead of CPET in CHF patients with T2DM [42].

There are some limitations in this study. First, we could not identify the causes of the lowered peak aerobic capacity in the CHF patients with T2DM, including cardiac and peripheral factors that determine the peak VO_2_. Second, our participants’ mean value of peak VO_2_ was higher than 14 mL/kg/min, a cut-off value of candidate of heart transplantation, indicating that most participants had a mild-to-moderate CHF. Third, some of the patients underwent echocardiography or blood testing on another day within 30 days before or after CPET, which might influence the data analysis, although all the participants were in stable condition. Finally, we could not eliminate patients with recovered or improved LVEF from the non-reduced LVEF cohort.

## Conclusions

T2DM is an independent predictor of the lower peak VO_2_ in CHF patients with non-reduced LVEF and in those with reduced LVEF. The comorbidity of T2DM has a negative impact on CHF patients’ exercise capacity, and the degree of impact is partly dependent on their LV systolic function. The prevention of T2DM is important not only for the reduction of cardiovascular risks but also for the maintenance of exercise capacity in HF management.

## Supplementary information

**Additional file 1. Table S1.** Baseline characteristics of the CHF patients with preserved LVEF (≥50%) or mid-range LVEF (40-49%). **Table S2.** Cardiopulmonary exercise testing parameters of the CHF patients with preserved LVEF (≥50%) or mid-range LVEF (40-49%). **Table S3.** Multivariable analysis for peak VO2 in the CHF patients with preserved LVEF (≥50%) or mid-range LVEF (40-49%).

## Data Availability

The datasets generated and/or analyzed during the current study are not publicly available due regulations of patient information to be released in public but are available from the corresponding author on reasonable request, after anonymization.

## Abbreviations

A: atrial systolic transmitral flow velocity
ACE: angiotensin converting enzyme
ARB: angiotensin receptor blocker
AT: anaerobic threshold
BNP: B-type natriuretic peptide
CHF: chronic heart failure
CI: chronotropic index
DPP4: dipeptidyl peptidase 4
CPET: cardiopulmonary exercise testing
E: peak early-diastolic transmitral flow velocity
e′: peak early-diastolic mitral annular velocity
eGFR: estimated glomerular filtration rate
HbA1c: glycohemoglobin A1c
HR: heart rate
LVEDD: left ventricular end-diastolic diameter
LVEF: left ventricular ejection fraction
MRAs: mineralocorticoid receptor antagonists
NYHA: New York Heart Association
RER: respiratory exchange ratio
SD: standard deviation
T2DM: type 2 diabetes mellitus
VCO_2_: carbon dioxide production
VE: minute ventilation
VO_2_: oxygen uptake
95% CI: 95% confidence interval
