# Supplementary material for: Type 2 diabetes is an independent predictor of lowered peak aerobic capacity in heart failure patients with non-reduced or reduced left ventricular ejection fraction
Source: Cardiovasc Diabetol. 2020 Sep 19;19:142. doi: 10.1186/s12933-020-01114-4 (PMC7502205; doi:10.1186/s12933-020-01114-4)
Supplement: Supplementary file 1 — Additional file 1. Table S1. Baseline characteristics of the CHF patients with preserved LVEF (≥50%) or mid-range LVEF (40-49%). Table S2. Cardiopulmonary exercise testing parameters of the CHF patients with preserved LVEF (≥50%) or mid-range LVEF (40-49%). Table S3. Multivariable analysis for peak VO2 in the CHF patients with preserved LVEF (≥50%) or mid-range LVEF (40-49%). [file 12933_2020_1114_MOESM1_ESM.docx]

**Table S1**. Baseline characteristics of the CHF patients with preserved LVEF (≥50%) or mid-range LVEF (40-49%)

|  | LVEF ≥50% | | | | LVEF 40-49% | | | |
| --- | --- | --- | --- | --- | --- | --- | --- | --- |
|  | **All**  **(n=73)** | **Diabetic**  **(n=14)** | **Nondiabetic**  **(n=59)** | ***P*-value** | **All**  **(n=58)** | **Diabetic**  **(n=20)** | **Nondiabetic**  **(n=38)** | ***P*-value** |
| Demographic findings: |  |  |  |  |  |  |  |  |
| Age, years | 57.5 ± 16.9 | 66.5 ± 9.9 | 55.4 ± 17.6 | 0.03 | 58.8 ± 14.1 | 60.2 ± 10.8 | 58.1 ± 15.6 | 0.49 |
| Male | 40 (55%) | 9 (64%) | 31 (53%) | 0.43 | 48 (83%) | 16 (80%) | 32 (84%) | 0.69 |
| BMI, kg/m^2^ | 23.5 ± 3.9 | 24.9 ± 4.7 | 23.2 ± 3.6 | 0.08 | 23.8 ± 4.3 | 25.4 ± 5.8 | 22.9 ± 2.9 | 0.05 |
| NYHA functional class: |  |  |  | 0.09 |  |  |  | 0.69 |
| I | 17 (24%) | 1 (7%) | 16 (27%) |  | 12 (21%) | 5 (25%) | 7 (21%) |  |
| II | 50 (68%) | 13 (93%) | 37 (63%) |  | 39 (67%) | 12 (60%) | 27 (71%) |  |
| III | 6 (8%) | 0 (0%) | 6 (10%) |  | 7 (12%) | 3 (15%) | 4 (11%) |  |
| Ischemic heart disease | 9 (12%) | 1 (7%) | 8 (14%) | 0.51 | 16 (28%) | 7 (35%) | 9 (24%) | 0.36 |
| Hypertension | 29 (40%) | 9 (64%) | 20 (34%) | 0.04 | 31 (53%) | 15 (75%) | 16 (42%) | 0.02 |
| Dyslipidemia | 19 (26%) | 4 (29%) | 15 (25%) | 0.81 | 29 (50%) | 16 (80%) | 13 (34%) | <0.01 |
| Atrial fibrillation | 20 (27%) | 9 (64%) | 11 (19%) | 0.01 | 13 (22%) | 5 (25%) | 8 (21%) | 0.73 |
| Echocardiographic findings: |  |  |  |  |  |  |  |  |
| LVEDD, mm | 50.8 ± 9.6 | 54.4 ± 8.8 | 49.9 ± 9.6 | 0.05 | 56.2 ± 6.8 | 57.6 ± 5.2 | 55.4 ± 7.4 | 0.18 |
| LVEF, % | 60.4 ± 7.3 | 57.7 ± 5.2 | 61.0 ± 7.6 | 0.06 | 43.9 ± 2.6 | 44.4 ± 2.6 | 43.7 ± 2.6 | 0.32 |
| E/A ratio^1)^ | 1.10 ± 0.40 | 1.26 ± 0.46 | 1.07 ± 0.39 | 0.36 | 1.04 ± 0.67 | 1.24 ± 1.27 | 0.98 ± 0.38 | 0.42 |
| Deceleration time^2)^, msec | 238.0 ± 63.2 | 204.9 ± 56.3 | 233.9 ± 64.2 | 0.19 | 244.0 ± 79.7 | 235.2 ± 82.9 | 247.8 ± 79.6 | 0.63 |
| E/e’^3)^ | 10.9 ± 3.8 | 13.3 ± 4.1 | 10.0 ± 3.4 | 0.02 | 9.4 ± 2.9 | 10.1 ± 3.0 | 9.2 ± 2.9 | 0.42 |
| Laboratory measurements: |  |  |  |  |  |  |  |  |
| Hemoglobin, g/dL | 13.1 ± 1.5 | 12.9 ± 1.6 | 13.2 ± 1.4 | 0.60 | 13.5 ± 1.8 | 13.7 ± 1.8 | 13.4 ± 1.8 | 0.80 |
| Serum creatinine, mg/dL | 0.9 ± 0.3 | 1.0 ± 0.3 | 0.9 ± 0.2 | 0.08 | 1.1 ± 0.4 | 1.1 ± 0.4 | 1.0 ± 0.4 | 0.31 |
| eGFR, mL/min/1.73 m^2^ | 66.4 ± 21.6 | 55.9 ± 19.4 | 68.9 ± 21.5 | 0.04 | 62.5 ± 22.8 | 57.8 ± 20.8 | 65.1 ± 23.7 | 0.26 |
| HbA1c, % | 5.7 ± 0.8 | 6.8 ± 0.9 | 5.5 ± 0.5 | <0.01 | 6.0 ± 0.8 | 6.7 ± 0.9 | 5.6 ± 0.3 | <0.01 |
| BNP, pg/mL | 199.5 ± 373.3 | 174.0 ± 139.5 | 205.8 ± 412.4 | 0.07 | 154.1 ± 153.8 | 207.6 ± 203.7 | 130.5 ± 122.3 | 0.32 |
| Medications: |  |  |  |  |  |  |  |  |
| ACE inhibitors or ARBs | 41 (56) | 11 (79) | 30 (51) | 0.06 | 53 (91) | 19 (95) | 34 (89) | 0.48 |
| β blockers | 34 (47) | 8 (62) | 26 (44) | 0.25 | 50 (86) | 19 (95) | 31 (82) | 0.16 |
| MRAs | 17 (24) | 3 (23) | 14 (24) | 0.96 | 24 (41) | 10 (50) | 14 (37) | 0.33 |
| Statins | 21 (29) | 2 (15) | 19 (32) | 0.23 | 27 (47) | 13 (65) | 14 (37) | 0.04 |
| Insulin | – | – | – | N.A. | 2 (3) | 2 (10) | – | N.A. |
| Metformin | 2 (3) | 2 (15) | – | N.A. | 2 (3) | 2 (10) | – | N.A. |
| DPP4 inhibitors | 1 (1) | 1 (8) | – | N.A. | 6 (10) | 6 (30) | – | N.A. |
| Sulfonylureas | 5 (7) | 5 (38) | – | N.A. | 2 (3) | 2 (10) | – | N.A. |

Data are means ± SD or n (%). A, peak velocity of mitral inflow during atrial systole; ACE, angiotensin converting enzyme; ARB, angiotensin II receptor blocker; BMI, body mass index; BNP, B–type natriuretic peptide; DPP4, dipeptidyl peptidase 4; E, peak velocity of mitral inflow during early diastole; e’, average of septal and lateral mitral annular early diastolic peak velocities; eGFR, estimated glomerular filtration rate; HbA1c, glycohemoglobin A1c; N.A., not applicable; LVEDD, left ventricular end–diastolic diameter; LVEF, left ventricular ejection fraction; MRAs, mineralocorticoid receptor antagonists; NYHA, New York Heart Association. 1) n=36, 5, 31 (all, diabetic, nondiabetic in LVEF ≥50%) and n=30, 7, 23 (all, diabetic, nondiabetic in LVEF 40-49%); 2) n=44, 9, 35 (all, diabetic, nondiabetic in LVEF ≥50%) and n=37, 11, 26 (all, diabetic, nondiabetic in LVEF 40-49%); 3) n=38, 10, 28 (all, diabetic, nondiabetic in LVEF ≥50%) and n=37, 10, 27 (all, diabetic, nondiabetic in LVEF 40-49%).

**Table S2**. Cardiopulmonary exercise testing parameters of the CHF patients with preserved LVEF (≥50%) or mid-range LVEF (40-49%)

|  | LVEF ≥50% | | | | | LVEF 40-49% | | | | |
| --- | --- | --- | --- | --- | --- | --- | --- | --- | --- | --- |
|  | **All**  **(n=73)** | **Diabetic**  **(n=14)** | **Nondiabetic**  **(n=59)** | **Unadjusted mean difference**  **(95% CI)** | **Adjusted mean difference**  **(95% CI)** | **All**  **(n=58)** | **Diabetic**  **(n=20)** | **Nondiabetic**  **(n=38)** | **Unadjusted**  **mean**  **difference**  **(95% CI)** | **Adjusted**  **mean**  **difference**  **(95% CI)** |
| Peak VO_2_,  mL/kg/min | 16.94 ± 5.38 | 15.70 ± 4.00 | 17.23 ± 5.64 | -0.76  (-236 to 0.83) | -0.34  (-1.70 to 1.02) | 16.78 ± 4.49 | 14.79 ± 3.75 | 17.83 ± 5.22 | -1.52*  (-2.70 to -0.34) | -1.43*  (-2.66 to -0.20) |
| Peak workload,  watts | 88.2 ± 41.3 | 82.0 ± 36.8 | 89.6 ± 42.4 | -3.8  (-16.1 to 8.5) | -1.8  (-10.6 to 7.1) | 94.7 ± 38.8 | 95.9 ± 47.2 | 94.1 ± 34.3 | 0.9  (-9.9 to 11.7) | 1.8  (-8.6 to 12.2) |
| Peak HR,  beats/min | 128.7 ± 30.8 | 128.0 ± 27.3 | 122.8 ± 31.8 | -0.4  (-9.6 to 8.8) | 5.9  (-3.1 to 14.8) | 117.7 ± 29.7 | 108.4 ± 29.4 | 122.6 ± 29.0 | -7.1  (-15.2 to 1.0) | -8.3  (-17.6 to 1.1) |
| Peak RER | 1.24 ± 0.13 | 1.16 ± 0.08 | 1.25 ± 0.13 | -0.05*  (-0.09 to -0.01) | -0.04*  (-0.08 to -0.01) | 1.25 ± 0.13 | 1.21 ± 0.14 | 1.27 ± 0.13 | -0.03  (-0.07 to 0.01) | -0.03  (-0.08 to 0.01) |
| ΔVO_2_/Δworkload | 7.26 ± 1.89 | 6.74 ± 1.49 | 7.39 ± 1.97 | -0.32  (-0.89 to 0.24) | -0.20  (-0.81 to 0.41) | 7.14 ± 2.32 | 6.82 ± 2.32 | 7.30 ± 2.34 | -0.24  (-0.89 to 0.41) | -0.13  (-0.71 to 0.45) |
| Peak O_2_ pulse, mL/beats | 8.14 ± 2.81 | 8.11 ± 2.96 | 8.15 ± 2.79 | -0.02  (-0.86 to 0.82) | -0.18  (-0.80 to 0.44) | 9.57 ± 3.11 | 10.10 ± 3.31 | 9.29 ± 3.00 | 0.40  (-0.46 to 1.27) | 0.54  (-0.17 to 1.26) |
| HR reserve,  beats/min | 58.8 ± 26.5 | 56.1 ± 26.6 | 59.4 ± 26.6 | -1.6  (-9.5 to 6.3) | 2.4  (-5.6 to 10.4) | 52.2 ± 25.7 | 44.2 ± 23.7 | 56.4 ± 26.0 | -6.1  (-13.1 to 0.8) | -6.9  (-15.2 to 1.4) |
| Chronotropic index | 0.65 ± 0.29 | 0.71 ± 0.36 | 0.63 ± 0.28 | 0.04  (-0.05 to 0.13) | 0.07  (-0.03 to 0.16) | 0.55 ± 0.29 | 0.47 ± 0.28 | 0.60 ± 0.28 | -0.06  (-0.14 to 0.01) | -0.07  (-0.17 to 0.02) |
| AT VO_2_,  mL/kg/min | 10.96 ± 3.01 | 10.4 ± 1.54 | 11.10 ± 3.26 | -0.37  (-1.26 to 0.53) | -0.43  (-1.36 to 0.50) | 10.50 ± 2.34 | 9.39 ± 1.51 | 11.08 ± 2.50 | -0.85*  (-1.46 to -0.24) | -0.85*  (-1.53 to -0.18) |
| Lowest VE/VCO_2_ | 34.1 ± 6.1 | 34.7 ± 4.4 | 34.0 ± 6.5 | 0.4  (-1.5 to 2.2) | 0.5  (-1.2 to 2.1) | 33.0 ± 5.9 | 33.6 ± 4.4 | 32.6 ± 6.6 | 0.5  (-1.2 to 2.2) | 0.4  (-1.4 to 2.1) |

Data are means ± SD. Mean difference between the diabetic and the nondiabetic in each CHF cohort is adjusted by age-squared, gender, hemoglobin, eGFR, LVEF, and log BNP. AT, anaerobic threshold; HR, heart rate; RER, respiratory exchange ratio; VCO_2_, carbon dioxide production VE, minute ventilation; VO_2_, oxygen uptake; 95% CI, 95% confidence interval. **P* < 0.05 vs. nondiabetics.

**Table S3**. Multivariable analysis for peak VO_2_ in the CHF patients with preserved LVEF (≥50%) or mid-range LVEF (40-49%)

|  | LVEF ≥50% | | LVEF 40-49% | |
| --- | --- | --- | --- | --- |
|  | **Adjusted mean difference**  **(mL/kg/min)**  **95% CI** | ***P*-value** | **Adjusted mean difference**  **(mL/kg/min)**  **95% CI** | ***P*-value** |
| Type 2 diabetes | -0.34  (-1.74 to 1.02) | 0.61 | -1.43  (-2.66 to -0.20) | 0.02 |
| LVEF, % | -0.10  (-0.27 to 0.08) | 0.29 | -0.15  (-0.64 to 0.33) | 0.53 |
| Log BNP | -0.46  (-2.63 to 1.71) | 0.67 | -0.04  (-1.81 to 1.72) | 0.96 |

In addition to the variables displayed, age-squared, gender, hemoglobin, and eGFR are included in the analysis.
